# Prospects for the Use of Amaranth Grain in the Production of Functional and Specialized Food Products

**DOI:** 10.3390/foods14091603

**Published:** 2025-05-01

**Authors:** Dana Toimbayeva, Saule Saduakhasova, Svetlana Kamanova, Amirsana Kiykbay, Sayagul Tazhina, Indira Temirova, Marat Muratkhan, Bakhyt Shaimenova, Linara Murat, Dina Khamitova, Gulnazym Ospankulova

**Affiliations:** Department of Food Technology and Processing Products, Technical Faculty, S.Seifullin Kazakh Agrotechnical Research University, Zhenis Avenue, 62, Astana 010000, Kazakhstan; bio.dana@mail.ru (D.T.); saule_aru@list.ru (S.S.); kamanovasveta@mail.ru (S.K.); tazhina_saya@mail.ru (S.T.); indira_t85@mail.ru (I.T.); marat@nwafu.edu.cn (M.M.); bshaymenova@mail.ru (B.S.); linaraazamatkyzy@mail.ru (L.M.)

**Keywords:** amaranth, functional nutrition, grain, bioactive components, therapeutic properties, chemical content

## Abstract

This review is dedicated to exploring recent advancements in the study of amaranth grain and presents research primarily on Amaranthus species such as *Amaranthus cruentus*, *Amaranthus hypochondriacus*, and *Amaranthus caudatus*, and to a lesser extent *Amaranthus hybridus*, *Amaranthus mantegazzianus*, *Amaranthus muricatus*, *Amaranthus tuberculatus*, *Amaranthus viridis*, *Amaranthus spinosus*, and *Amaranthus tenuifoliu*. Amaranth (*Amaranthus* spp.) is a promising, high-yield pseudocereal crop with significant commercial potential for developing functional food products. It contains a wide range of bioactive compounds, including squalene, tocopherols, phenolic compounds, phytates, and vitamins, which possess important physiological properties. Amaranth grain is characterized by high levels of starch, proteins, minerals, and dietary fiber. Moreover, amaranth proteins are distinguished by a balanced amino acid composition and exhibit greater resistance to external factors compared to animal-derived proteins. Grains of amaranth are free of gliadin, making it a valuable nutritional source for individuals with celiac disease, an immune-mediated disorder. Unlike traditional cereals, where prolamins and glutelins dominate the protein composition, the proteins of pseudocereals like amaranth primarily consist of albumins and globulins. The processing methods of amaranth grain influence their quantitative and qualitative composition, often significantly improving their physicochemical, antioxidant, functional, and rheological properties. This work provides a detailed analysis of amaranth’s chemical composition and bioactive components, along with its evaluation of therapeutic and preventive properties. Amaranth protein fractions (albumin, globulin, and glutelin) and squalene exhibit increased antioxidant activity, contributing to notable resistance to radiation and X-ray exposure. Bioactive compounds such as phytol, α-tocopherol, and a lunasin-like peptide (AhLun) with potential anticancer properties have also been identified in amaranth. Furthermore, six bioactive peptides were isolated and identified from amaranth, which, according to predictive models, demonstrate a high capacity to inhibit angiotensin-converting enzyme (ACE) activity, suggesting potential hypotensive effects. Certain amaranth peptides are considered promising functional food ingredients for the prevention and comprehensive treatment of conditions such as diabetes, inflammatory bowel diseases, hypercholesterolemia, cardiovascular diseases, and obesity. *Amaranthus* spp. and its processed products hold significant interest for the development of innovative food products, contributing to the expansion of their range and enhancement of nutritional value.

## 1. Introduction

Amaranth (*Amaranthus* spp.) is frequently regarded as a promising alternative to traditional cereal crops [1]. Unlike most traditional cereal crops, amaranth is characterized by a balanced amino acid composition, including all essential amino acids, the absence of gluten in its grain and high levels of dietary fiber, antioxidants (polyphenols, squalene, α-tocopherol), macro- and microelements (iron, calcium, magnesium, zinc), phytochemical compounds, and bioactive peptides [2,3,4,5,6,7].

In the mid-1970s, the U.S. National Academy of Sciences [8] identified three species of amaranth—*A. Caudatus*, *A. cruentus*, and *A. hypochondriacus*—traditionally cultivated in Central America and Mexico which are essential food sources with substantial potential for further breeding. The likely wild relatives or ancestors of these species are *A. powellii* and *A. hybridus*, both of which are widely distributed across Mexico [9].

Currently, the amaranth species used for food applications include *A. cruentus*, *A. caudatus*, and *A. hypochondriacus* [10]. These species are actively cultivated in North America (the USA and Canada), South America (Guatemala, Peru, and Argentina), Europe (Germany and France), Asia (India and China), and Africa (Ethiopia). This pseudocereal crop is highly tolerant to adverse environmental conditions: it can thrive in saline and alkaline soils, withstand high temperatures, grow at high altitudes, and endure periods of water scarcity [11,12]. Furthermore, amaranth is considered an environmentally sustainable crop, as its natural resistance to pests allows it to be grown without the need for chemical treatments or fertilizers.

In terms of nutritional content, amaranth seeds, whether in their wild or cultivated forms, have a notably high protein content compared to other cereal crops [13]. Unlike traditional cereals such as maize and rice, which predominantly concentrate protein in the endosperm, amaranth stores the majority of its protein (up to 65%) in the germ and seed coat [14].

The protein profile of amaranth is mainly composed of easily digestible albumins and globulins [15,16]. From a nutritional perspective, a key feature of amaranth’s protein composition is the presence of all essential amino acids, which the human body cannot synthesize and must obtain from external sources. The isoleucine, leucine, and lysine are particularly abundant, with the lysine content in amaranth being twice as high as in traditional crops [2]. Bioactive peptides derived from amaranth proteins exhibit diverse physiological effects, including anticholesterolemic, antihypertensive, antioxidant, and antithrombotic activities [17].

The carbohydrate composition of amaranth seeds is also actively studied. The starch content of amaranth exceeds 60%, with an amylose fraction ranging from 4.7% to 12.5% [18,19,20]. The total concentration of mono- and oligosaccharides (glucose, fructose, sucrose, and raffinose) in dry matter ranges from 3% to 4%, with sucrose being the predominant component, at a content twice that found in the grains of traditional cereals [21].

Amaranth seeds are rich in dietary fiber, which has hypotriglyceridemic effects that aid in regulating the metabolism of both saturated and unsaturated fatty acids [4]. The lipid profile of amaranth is composed of triacylglycerols (TAGs), phospholipids, squalene, and fat-soluble vitamins, primarily tocopherols, which are the main components of the lipophilic fraction of the seeds [22].

Despite the mentioned advantages, amaranth is still rarely utilized, particularly as a cereal crop, despite its significant potential in the food industry [5,23,24]. Amaranth and its processed derivatives play a crucial role in the development of innovative food products, broadening their variety [25]. Thermal and biological processing of amaranth enhances its food acceptability and nutritional profile while also increasing the antioxidant activity and bioavailability of bioactive compounds [26,27].

This review examines the latest developments in the study of amaranth grain, focusing on its chemical composition and bioactive components, and highlights its therapeutic and preventive properties. These include antioxidant effects, antihypertensive benefits, anti-obesity properties, and antimicrobial, antitumor, hypocholesterolemic, immunomodulatory, antidiabetic, and anti-inflammatory effects, as well as its influence on the intestinal microflora. Significant attention is also given to amaranth grain processing technologies aimed at extracting and concentrating its bioactive compounds to develop innovative functional food products.

## 2. Chemical Composition

Amaranth grain is a pseudocereal crop with exceptional nutritional and biological potential. It is abundant source of high-quality protein, enriched with essential amino acids vital for human nutrition [28,29,30]. Additionally, it has a substantial lipid content, primarily consisting of unsaturated fatty acids, such as linoleic, oleic, and palmitic acids [31,32]. Its carbohydrates are easily digestible [33], and it provides dietary fiber [34], as well as a broad spectrum of vitamins and minerals [5]. Furthermore, amaranth is a valuable source of numerous bioactive compounds, including phenolic acids, flavonoids, squalene, and other antioxidants [35], which exhibit pronounced functional properties.

**Figure 1:**
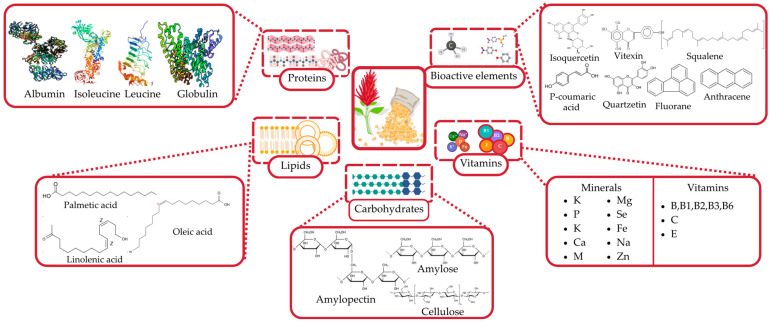
Chemical composition and bioactive compounds of amaranth grain.

Figure 1 provides a comprehensive diagram of the chemical composition of amaranth grain, highlighting its primary nutrient groups and bioactive compounds. The illustration clearly depicts the protein, lipid, carbohydrate, vitamin, and mineral components, alongside a diverse array of biologically active substances, such as phenolic compounds, flavonoids, and squalene.

### 2.1. Proteins, Essential Amino Acids, and Bioactive Peptides

Amaranth grains are known for their high protein content, which ranges from 13.1% to 21.5% in cultivated species [13,28,29,36,37]. Amaranth proteins are particularly rich in lysine- and sulfur-containing amino acids, which effectively form structured gel networks through inter-protein SH/SS (sulfhydryl/disulfide) exchange reactions [30,38].

The protein composition of amaranth consists of various fractions [30]. Primarily, amaranth grain proteins are composed of albumin (46% to 65%) and globulins (approximately 20%), which determine the structural and physicochemical properties of amaranth concentrates and isolates [15,16]. Increasing the content of soluble proteins in the protein complex is known to enhance its nutritional value. Albumin mainly contains tryptophan, threonine, and lysine.

Besides albumin and globulins, amaranth proteins also contain 25% to 30% glutelins and 12% alcohol-soluble prolamines, which do not contain essential amino acids [22,39]. Other studies indicate that glutelins are the major protein in amaranth, making up 42.5% to 46.5% of the total protein, while albumin accounts for 19% to 23% [40,41]. These differences in results may be due to geographical location and the variety of amaranth, as well as fractionation methods and protein variability [42,43].

Proteomic studies on amaranth seeds from *A. hypochondriacus* and *A. cruentus* are gaining importance, offering new approaches to improve amaranth production [44,45,46,47,48]. Research indicates that amaranth globulins are composed of 11S and 7S fractions. The 11S fraction includes globulin 11S and its hydrophobic isoform P-globulin, which consists of two subunits with a molecular mass similar to that of 11S globulin, as well as a polypeptide with a molecular mass of 56 kDa [47]. A study [49] suggests that amaranth glutelins might represent polymeric forms of globulins. In another study [44], a two-dimensional gel electrophoresis (2-DE) map of *A. cruentus* L. cv Amaranteca seeds was created, and proteins were identified from it. One of the identified proteins was the late embryogenesis abundant (LEA) protein, which was cloned and characterized. The AcLEA protein exhibited unique structural features, thereby classifying it as a new protein within the LEA 5 group. The identified proteins are associated with stress and defense responses, as well as metabolic and oxidative reduction processes.

A comparative proteomics analysis of wild-type and cultivated amaranth species revealed that granule bound starch synthase I (GBSSI) and seed storage proteins (SSPs) were the most abundant proteins exhibiting differential accumulation in both hydrophilic and hydrophobic fractions. Both proteins were predominantly identified in the hydrophobic fractions, indicating their high hydrophobicity and tendency to aggregate. However, their lower presence in the hydrophilic fraction suggests significant variability in their physicochemical properties, such as solubility [48].

According to a study [48], SSPs account for 38% of protein variation, which is defined by three main proteins: 11S globulin, Vicilin-like SSP (018839), and Vicilin-like SSP At2g18540 (010140).

The high biological value of amaranth protein is attributed to its balanced amino acid composition [3]. The amino acid content in amaranth proteins differentiates depending on the plant variety. For instance, the lysine content in the proteins of *A. caudatus*, *A. hybridus*, *A. cruentus*, and *A. hypochondriacus* ranges from 0.73% to 0.84%, and tryptophan ranges from 0.18% to 0.28% [50]. The amino acid profile of amaranth is closer to that of legumes than to cereals, except for sulfur-containing amino acids, which are more abundant in amaranth (4.5–5.6%) compared to legumes (1.4%) [22,29,51]. Additionally, the amounts of cysteine and methionine in amaranth protein are higher than those found in cereals and legumes [52]. The proportion of essential amino acids (excluding tryptophan) in amaranth ranges from 43% to 49% [52]. A summary of essential amino acids in amaranth seeds is presented in Table 1.

Table 1 illustrates that the content of specific amino acids in amaranth seeds varies within distinct ranges. The concentrations of leucine, phenylalanine, threonine, methionine, and isoleucine show relatively minor variations, while valine, lysine, and histidine exhibit more significant changes. Several factors may contribute to this variability. First, the studies examined different amaranth species, such as *A. hypochondriacus* L., *A. cruentus*, and varieties like Annapurna and Durga. Second, the plants were grown in diverse climatic regions, including Mexico, India, Portugal, and Romania, which likely influenced the grains’ biochemical composition. Third, variations in analytical methods, such as improved methodologies and differences in grain preparation procedures, also explain the noted discrepancies.

It is important to mention that the amino acid content data reported by Awasthi et al. [53] and Motta et al. [54] are closely aligned. Additionally, for several amino acids, these values fall within the average range when compared to findings from other studies [5,55]. This consistency suggests that these results can be considered conditionally representative of amaranth’s amino acid profile.

The lysine content in amaranth far exceeds that in most cereal crops, ranging from 55 mg/g to 65 mg/g or higher, positioning amaranth alongside soybeans as an alternative source of this essential amino acid for adults [2,8,56,57]. Insufficient lysine consumption may result in compromised immune function, decreased blood protein levels, and hindered cognitive and physical growth in children [58]. Enriching staple foods like rice with lysine offers a promising strategy to address malnutrition, especially in at-risk communities [59].

Additionally, the high levels of arginine and histidine, which are essential amino acids for children and infants, make amaranth a valuable component in infant nutrition [60,61].

Although amaranth protein contains lower amounts of leucine, this is not a significant limitation, as leucine is available in sufficient quantities in other common cereal crops [61,62].

Amaranth’s albumins and globulins contain higher amounts of amino acids, such as lysine, methionine, cysteine, and histidine, whereas its prolamins are characterized by higher levels of glutamic acid and proline [54,63]. Bressani et al. [42] reported that prolamins have high lysine content, while glutelin and glutelin-like protein fractions contain lower levels of this amino acid.

Furthermore, the digestibility of amaranth proteins, which ranges from 79% to 97%, is comparable to that of milk casein protein (100%) [64,65,66].

Bioactive peptides, molecules with potential biological activity, have a significant impact on human health and represent an alternative approach to preventing various metabolic diseases. These peptides exhibit a wide range of activities, high biospecific activity, lower allergenicity, and structural diversity. To date, peptides with notable potential in antioxidant, antihypertensive, antithrombotic, hypocholesterolemic, immunomodulatory, antidiabetic, and anti-inflammatory activities have been isolated and identified from proteins and their hydrolysates in various amaranth varieties [61,67,68,69].

### 2.2. Fats

Amaranth grains typically contain 6–9% fat [31,70], although the oil content can reach as high as 19.3% in the seeds of *A. spinosus* and *A. tenuifolius* [71]. Research indicates that the oil content of amaranth varies depending on the species, cultivar, agricultural techniques, and geographical location of cultivation [72].

The high fat content of amaranth makes it more susceptible to oxidation. Kinetic studies on oxidation reactions have shown that a small amount of energy is sufficient to initiate oxidation in amaranth oil [73]. However, amaranth oil also exhibits a better resistance to oxidation, which enhances its storage stability [74].

The lipid content and profile in amaranth seeds are influenced not only by species and variety but also by growth conditions and extraction methods [75,76,77]. The primary fatty acids found in the oils of different amaranth seed varieties include linoleic acid (40.1–53.5%), oleic acid (14.8–31.3%), palmitoleic acid (15.7–23.19%), stearic acid, and vaccenic acid [31,32]. The predominance of linoleic acid is particularly notable, as it is an essential fatty acid that the human body cannot synthesize. Stearic, linolenic, arachidonic, and myristoleic acids were also present but in lower concentrations [72,78].

The total saturated fatty acids in amaranth oil ranged from 16.54% to 27.2%, while unsaturated fatty acids accounted for 72.7% to 83.45% [55,78]. The molar ratio of saturated to unsaturated fatty acids was 0.37 [78], indicating a balanced fatty acid composition that facilitates better absorption by the human body. According to various sources, the phospholipid content in amaranth oil ranged from 3.60% to 5.0% [79,80,81]. Opute et al. [80] found that 3.6% of phospholipids contained 13.3% cephalin, 16.3% lecithin, and 2.8% phosphoinositol.

Amaranth has also been identified as a rich source of eicosapentaenoic acid, with a composition similar to that of wheat germ oil [82]. The presence of unsaturated fatty acids in amaranth oil may provide health benefits, as linoleic acid helps lower serum cholesterol and low-density lipoprotein (LDL) levels. In contrast, oleic acid, although not significantly affecting LDL, moderately increases high-density lipoprotein (HDL) levels [83]. This distinctive fatty acid profile may reduce the risk factors associated with cardiovascular disease [84].

### 2.3. Carbohydrates and Dietary Fibers

The primary component of amaranth seeds is polysaccharides, with starch being the predominant one, accounting for 61% to 75% of the total content [34,85]. The amylose content in amaranth starch is lower than that in traditional cereal starches, ranging from 4.7% to 12.5% [18,20]. This characteristic of amaranth starch affects its physicochemical properties [19]. The amylopectin content in the starch can reach up to 97.9% [86]. Notably, the ratio of short-chain amylopectin to long-chain amylopectin is 2.2–2.6, which is slightly lower than that of corn starch. The digestion time of amaranth starch by amylase (3 h) is also shorter than that of corn starch [87].

The content of low-molecular-weight carbohydrates in the seeds of *A. cruentus* and *A. caudatus* ranges as follows (g/100 g): sucrose (0.58–0.75), glucose (0.34–0.42), fructose (0.12–0.17), maltose (0.24–0.28), raffinose (0.39–0.48), stachyose (0.15–0.13), and inositol (0.02–0.04) [33]. According to [21], sucrose is the predominant free sugar, with a content of about 2 g/100 g.

The fiber content in amaranth seeds varies between 2.2% and 8.1%, with soluble dietary fiber comprising 14% of the total fiber content [88,89]. The total dietary fiber content in amaranth varies from 8.6% to 15.8% per 100 g of dry matter [90,91]. In another study, these values were higher, ranging from 12.0% to 20.6% [92].

Amaranth grains are rich in diverse dietary fibers [93]. Analysis of the monosaccharide composition of dietary fibers extracted from amaranth seeds shows that they are primarily composed of galacturonic acid, arabinose, xylose, glucose, and galactose. Linkage analysis further indicates that the predominant fraction of both soluble and insoluble dietary fiber in amaranth is classified as a pectin polysaccharide [94]. Xyloglucans represent the second most abundant type of dietary fiber found in whole amaranth grain.

In the study by Vidal Torres et al. [95], the percentages of insoluble, soluble, and total dietary fiber in the seeds of four amaranth varieties were found to be 7.95–11.20%, 4.75–6.25%, and 12.7–16.98%, respectively.

Additionally, Hozová et al. [96] reported that amaranth seeds contain more than 25% water-insoluble β-(1,3)-D-glucan, which is less than in oats but higher than in other grains and pseudocereals. Other studies have noted that the β-glucan content does not exceed 0.5%, with the highest proportion found in *A. hybridus* and *A. hypochondriacus* × *A. hybridus* [97,98]

The average content of resistant starch in 25 amaranth varieties was 12.4 ± 2.2 g/kg of dry matter [99]. It is well known that insoluble dietary fiber helps normalize gastrointestinal function by increasing stool volume and reducing transit time through the large intestine.

### 2.4. Minerals and Vitamins

Amaranth grains are rich in essential minerals and vitamins, making them a valuable food source for nutrition [5]. A study by De Bock et al. [85] analyzed the mineral content in coarse ground amaranth flour, revealing high levels of phosphorus (P) at 5011 mg/kg dry matter, potassium (K) at 4740 mg/kg, magnesium (Mg) at 2755 mg/kg, and calcium (Ca) at 1930 mg/kg. The sodium (Na) content was 75.7 mg/kg, iron (Fe) was 82.6 mg/kg, manganese (Mn) was 30.0 mg/kg, and zinc (Zn) was 5.5 mg/kg. Additionally, Liu et al. [100] reported a selenium (Se) content of 18.7 mg. Zinc plays a critical role in stabilizing the immune system, iron helps alleviate anemia, and magnesium and manganese are essential for infant nutrition [85].

Other studies, such as those by Bhat et al. [101], have reported lower mineral levels in amaranth grains, with a particularly low sodium content of 1 mg/kg in pseudocereals [102].

Regarding vitamins, amaranth grains contain vitamin B1 (0.12 mg/100 g), vitamin B2 (0.20 mg/100 g), vitamin B3 (0.92 mg/100 g), and vitamin C (4.20 mg/100 g). A 100 g serving of amaranth flour provides 53% of the recommended daily intake of vitamin B6. A study by Oh et al. [103] reported similar values for vitamin B2 content in amaranth grains (0.431 ± 0.023 µg/g crude weight), with vitamin B6 content at 0.184 ± 0.003 µg/g crude weight.

Amaranth oil is also rich in vitamin E (705–829 mg/kg), with δ-tocopherol being the predominant component [31]. The content of various tocopherols in amaranth oil samples extracted by supercritical CO_2_ extraction, solvent extraction, and cold pressing were as follows: for α-tocopherol (22.8, 23.9, and 23.0 mg/100 g oil), β-tocopherol (53.0, 34.0, and 49.0 mg/100 g oil), δ-tocopherol (41.1, 31.8, and 40.3 mg/100 g oil), and γ-tocopherol (14.8, 11.2, and 15.6 mg/100 g oil) [76]. The tocopherol content in commercially available 100% cold-pressed amaranth oil, as reported by Ogrodowska et al. [104], is slightly different: α-tocopherol (21.26 mg/100 g), β-tocopherol + γ-tocopherol (20.85 mg/100 g), and δ-tocopherol (9.34 mg/100 g).

Using normal-phase high-performance liquid chromatography with fluorescence detection, Lehmann et al. [105] investigated the tocopherol composition in seeds of thirteen amaranth varieties (*A. cruentus* L., *A. hypochondriacus* L.). The most abundant tocopherols were α-tocotrienol (2.97 to 15.65 mg/kg seed), β-tocotrienol (5.92 to 11.47 mg/kg seed), and γ-tocotrienol (0.95 to 8.69 mg/kg seed), while some *A. cruentus* samples contained δ-tocotrienol (0.01 to 0.42 mg/kg seeds).

Tocopherols and tocotrienols exhibit antioxidant properties and serve critical biological functions in both plant cells and the human body. They are widely used for the prevention and treatment of cardiovascular diseases, neurodegenerative disorders, cancer, atherosclerosis, hyperlipidemia, and osteoporosis [106,107].

### 2.5. Other Bioactive Components

To date, a considerable amount of information is available on various microcomponents of amaranth grain, including secondary metabolites that possess functional properties. These include phenolic compounds, flavonoids, squalene, and carotenoids, all of which exhibit antioxidant, antibacterial, anti-inflammatory, antiviral, and anticancer activities and help regulate lipid metabolism [22,35].

A study on *A. mantegazzianus* [6] identified several phenolic compounds, including flavonoids such as quercetin, isoquercetin, and kaempferol, as well as phenolic acids like p-coumaric acid and 4-hydroxybenzoic acid. Gallic acid, 3,4-dihydroxybenzoic acid, and p-hydroxybenzoic acid were detected in amaranth seeds at concentrations ranging from 11.0 to 440, 4.7 to 136, and 8.5 to 20.9 mg/kg dry seed, respectively [108,109,110]. Bound ferulic acids, such as trans-ferulic acid (620 mg/kg) and cis-ferulic acid (203 mg/kg), were also detected in amaranth seeds following alkaline and enzymatic hydrolysis [111]. Compared to other phenolic acids found in *A. viridis* seeds, ferulic acid (11.46 mg/100 g) and p-hydroxybenzoic acid (10.92 mg/100 g) exhibited the highest concentrations [7]. Additionally, vanillic, caffeic, synapic, and cinnamic acids were detected but in lower concentrations [22].

The rutin content in amaranth seeds ranged from 0.08 mg/g in seeds to 24.5 mg/g in leaves [112]. Manyelo et al. [113] reported a rutin content of 252 mg/kg in mature *A. cruentus* seeds. In *A. hypochondriacus* seeds, the rutin content was higher, ranging from 310 to 508 mg/kg of dry matter [114]. This difference may be attributed to the distinct amaranth species used in these studies.

Vitexin (410 mg/kg) and isovitexin (266 mg/kg) were also detected in the seeds of *A. cruentus* v. Rawa, with a total flavonoid content of 676 mg/kg [109].

The lipid fraction of amaranth seeds contains up to 11% squalene, a key precursor of triterpenes and steroids, including sterols and their derivatives, which are used to treat atherosclerosis [77,115,116]. Amaranth is one of the richest plant sources of squalene [22,29,117,118]. Squalene offers significant cardioprotective benefits by lowering levels of low-density lipoprotein cholesterol and triglycerides, coupled with its robust antioxidant and anti-inflammatory properties [119]. Furthermore, it supports metabolic health by reducing blood glucose and liver triglyceride levels and inducing insulin production [120].

Despite the high squalene content in amaranth (5942 mg/100 g of grain fat), its commercial use remains limited, as most commercial squalene production is derived from olive oil (564 mg/100 g) [121,122].

In addition to squalene, a study by León-Camacho et al. [123] identified several saturated (13) and unsaturated (10) hydrocarbons with one double bond (C21–C33) in *A. cruentus* crude oil, with concentrations ranging from 1.91 to 64.9 ppm. In another study by Ciecierska et al. [124], the total content of polycyclic aromatic hydrocarbons in cold-pressed amaranth oil was quantified at 101.6 mg/kg, with phenanthrene, anthracene, fluoranthene, and pyrene being the most significant.

Amaranth oil contains approximately 20% free sterols, with 80% of the sterols being esterified [123]. Fifteen sterols were identified in *A. cruentus* oil [123], with the most important being 24-methylene-cholesterol, campesterol, stigmasterol, D7-campesterol, clerosterol, β-sitosterol, D5-avenasterol, D7-stigmasterol, and D5-avenasterol. Their concentrations (% of total sterols) were 0.3%, 1.6%, 0.9%, 24.8%, 42.0%, 1.3%, 2.0%, 15.2%, and 11.9%, respectively. Research on six amaranth species (*A. cruentus*, *A. hybridus*, *A. hypochondriacus*, *A. muricatus*, *A. tuberculatus*, and *A. viridis*) reveals that β-sitosterol, brassicasterol, campesterol, and stigmasterol are the predominant sterols found in amaranth oil [125].

Amaranth seeds also contain carotenoids such as lutein (0.355–0.444 mg/100 g) and zeaxanthin (0.014–0.030 mg/100 g), which are essential nutrients not synthesized independently in the human body [126].

Thus, amaranth represents a promising source of bioactive antioxidants that can be utilized in the development of functional food products [127].

## 3. Therapeutic and Preventative Properties of Amaranth

Currently, considerable attention is paid to the studies of grain amaranth in terms of its therapeutic and prophylactic properties, with an emphasis on antioxidant, antihypertensive, antimicrobial, antitumor, hypocholesterolemic, immunomodulatory, antidiabetic, and anti-inflammatory activities. The effect of amaranth on the intestinal microflora population is also actively studied, and promising areas of its use in clinical practice are identified.

Clinical studies of the effects of amaranth grains on human health are summarized in Table 2 [128,129,130,131,132,133,134,135,136,137].

**Figure 2:**
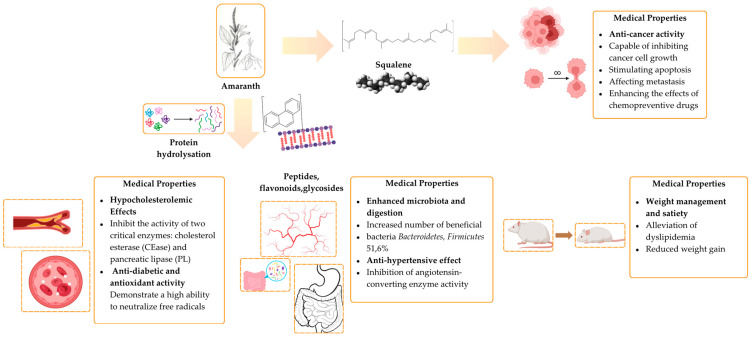
The complex effects of amaranth components on human health which highlight its potential for the creation of functional foods and nutraceuticals.

The multifaceted effects of amaranth’s bioactive components on human health, highlighting its significant potential as a raw material for functional foods and nutraceuticals is illustrated in Figure 2. The figure showcases key amaranth compounds (squalene, protein hydrolysates, peptides, flavonoids, and glycosides) and their physiological effects, including antioxidant, hypolipidemic (hypocholesterolemic), antihypertensive, antidiabetic, and antitumor activities, as well as modulation of the microbiota and body weight regulation [134,136,137,138,139,141,142,146].

### 3.1. Enhancing Gut Microbiota and Digestion

Dysbiosis, a disruption of normal gut function, is associated with various diseases, including ulcerative colitis, Crohn’s disease, and irritable bowel syndrome (IBS) [151]. It is also implicated in metabolic disorders such as cancer, obesity, diabetes, and cardiovascular diseases [151]. As a result, there has been growing interest in plant-based prebiotics, with amaranth emerging as a promising food source that may promote health and reduce the risk of metabolic diseases [152].

The functional characteristics of amaranth are mainly due to its levels of soluble and insoluble dietary fibers [88]. These fibers greatly affect the gut microbiota by supporting the proliferation of helpful bacteria, including *Bacteroides*, *Parabacteroides*, *Lactobacillus*, *Bifidobacterium*, *Lachnospiraceae*, and *Ruminococcaceae*, while decreasing the abundance of possibly harmful taxa, such as *Fusobacterium*, *Enterobacterium*, and *Porphyromonadaceae*. These impacts are supported by several research studies [129,153,154,155].

The soluble fiber fraction in amaranth consists of pectin, uronic acids, and high-molecular-weight carbohydrates which have residues of glucose, arabinose, xylose, mannose, and galactose. These compounds exert their physiological effects through interconnected mechanisms. During bacterial fermentation in the colon, they facilitate the production of short-chain fatty acids (SCFAs), which regulate intestinal pH and maintain the integrity of the mucosal barrier. Additionally, soluble fibers modulate insulin and glucagon secretion, influencing lipid metabolism in the liver and peripheral tissues [156,157]. They also increase the viscosity of intestinal contents, slowing lipid absorption, binding bile acids, and enhancing cholesterol catabolism, thereby exerting a hypolipidemic effect.

In contrast, the insoluble fiber fraction primarily has mechanical effects. It stimulates peristalsis, accelerates the transit of intestinal contents, and increases fecal bulk, thereby improving gastrointestinal motility [88,156,158,159].

In a study by Calva-Cruz et al. [138], researchers conducted a pilot trial to evaluate the effect of popped amaranth consumption on the composition of the gut microbiota in children with low height for their age. It was found that consumption of popped amaranth contributed to the restoration of gut microbiota. A decrease in the relative abundance of bacteria associated with inflammatory processes and colitis (*Alistipes putredinis*, *Bacteroides coprocola*, *Bacteroides stercoris*) and an increase in the abundance of bacteria associated with health and longevity (*Akkermansia muciniphila*, *Streptococcus thermophilus*) were observed [138].

Amaranth affects the microbiota at the taxonomic level by reducing the abundance of *Actinobacteriota* (by up to 9%) while promoting an increase in the proportion of *Bacteroidetes* and *Firmicutes*, which together reach a combined abundance of 51.6%. This raw material contributes to the recovery of the *Verrucomicrobiota* population, which is often deficient in children suffering from nutritional deficiencies. The study observed a significant increase in propionic acid and a trend toward increased acetic and butyric acids in the undernutrition group after amaranth consumption (UNA group). SCFAs lower gut pH, creating an environment favorable for beneficial bacteria while inhibiting pathogens. Butyrate, in particular, is a primary energy source for colonocytes, promoting gut barrier integrity and reducing inflammation [138]. Functional analysis has demonstrated the activation of metabolic pathways for acetic, butyric, and propionic acids, which promotes the synthesis of beneficial short-chain fatty acids (SCFAs) [138]. The study was conducted on a limited sample pool: 21 children comprised the control group, while 7 children were included in the group showing signs of stunting. The intervention lasted three months, which may not be sufficient to observe the long-term effects of popped amaranth consumption. Despite the positive findings, the authors highlight the need for further research, including randomized controlled clinical trials with a larger sample size. Such studies would allow for a more reliable assessment of the effects of amaranth on gut microbiota and its potential long-term health impacts in children [138]. According to a study by Sanna et al. [160], soluble dietary fiber is fermented by anaerobic microorganisms in the colon to produce SCFAs, which reduce the risk of obesity and diabetes. SCFAs play a critical role in regulating host metabolism, the immune system, and cell proliferation [161].

### 3.2. Antioxidant Activity of Amaranth

Amaranth is rich in bioactive compounds that confer pronounced antioxidant properties, contributing to the maintenance and improvement of the body’s functional status. The structural conformation, degree of hydrolysis, hydrophobicity, and amino acid composition of peptides are key factors that contribute to the antioxidant activity of amaranth [139]. Studies have demonstrated that amaranth protein hydrolysates (322.5–525.2 μmol TE/μg) exhibit a stronger ability to neutralize free radicals compared to native amaranth protein isolate (202.7 μmol TE/μg) [162].

Mudgil et al. [162] observed significant antioxidant activity in peptides derived from amaranth 11S-globulin. Similar findings were reported by Sandoval-Sicairos et al. [146], who identified high antioxidant activity in peptides with molecular weights of 3–10 kDa derived from germinated amaranth seeds [146]. Sprouted amaranth flour, when optimized, contains higher levels of protein, antioxidants, and dietary fiber compared to raw amaranth flour [27].

Amaranth proteins, including albumin, globulin, and glutelin, exhibit varying antioxidant activities, with hydrolysates of these fractions demonstrating higher efficacy. The amino acid methionine, which is involved in various mechanisms of free radical neutralization, enhances the overall antioxidant activity of proteins, underscoring the importance of amino acid composition in counteracting oxidative processes [139].

Phenolic compounds significantly contribute to amaranth’s antioxidant and gastroprotective activity. Both free and bound forms of flavonoids (rutin, isoquercetin, quercetin, kaempferol) and phenolic acids (p-coumaric acid, 4-hydroxybenzoic acid) were identified in the composition of amaranth flour and protein isolate. About 15% of phenolic compounds are bound with protein fractions, emphasizing the importance of intermolecular interactions in the structure of the food matrix [6]. Modeling of gastrointestinal digestion processes showed that during enzymatic hydrolysis, a release of previously bound polyphenols occurs, which is accompanied by an increase in antioxidant activity determined using ORAC (Oxygen Radical Absorbance Capacity) and ABTS (2,2′-Azino-bis (3 ethylbenzothiazoline 6 sulfonic acid) radical cation decolorization assay) methods. Catechin, quercetin, and kaempferol persist after simulated digestion, remaining biologically active and potentially more bioavailable for absorption [6].

The most significant contribution to antioxidant activity is made by derivatives of hydroxycinnamic acids (hydroxycinnamic acids), in particular ferulic, sinapic, and p-coumaric acids (p-coumaric, sinapic, cinnamic, and ferulic acids), which have a high correlation with DPPH (2,2-diphenyl-1-picrylhydrazyl radical) and MCA (Metal Chelating Activity). Ferulic acid showed maximum correlation with MCA, emphasizing its key role in preventing oxidation processes [7]. Popoola et al. [7] states that germination promotes an increase in both phenolic acids and flavonoids, including rutin and quercetin diglycoside. In addition, squalene, found in significant amounts in amaranth grains, contributes to the body’s resistance to radiation and X-rays [144]. The combined presence of squalene in amaranth oil with essential fatty acids, vitamins, and non-saponifiable components significantly enhances its antioxidant activity. In addition to squalene, amaranth oil contains other biologically active substances with antioxidant properties, particularly tocopherols, phytosterols, essential unsaturated fatty acids (olive and linoleic), and various non-saponifiable compounds. The synergetic interaction of all these components makes amaranth oil a powerful source of antioxidants, which determines its high value for the food industry [163]. Squalene and tocopherols are the most important bioactive components of the lipophilic fraction of amaranth. During the extraction of the lyophilic fraction of amaranth seeds by different methods, Kraujalis et al. [75] found that combinations of different components, including squalene and tocopherols, lead to a significant enhancement of the overall antioxidant activity, which is of important interest to the food and pharmaceutical industries [75].

Thus, based on the considered data, the antioxidant potential of amaranth is due to the complex interaction of different groups of biologically active compounds. In particular, ferulic acid and flavonoids (kaempferol, rutin, and quercetin) in combination with lipophilic components (squalene, tocopherols, and other non-saponifiable substances) make a significant contribution to the neutralization of free radicals. At the same time, protein fractions and peptides of amaranth formed during enzymatic hydrolysis demonstrate a high capacity for antioxidant activity due to their amino acid constitution and structural features. The content and bioavailability of all these compounds significantly depend on the applied methods of raw material processing, which makes amaranth a promising functional ingredient for the food and pharmaceutical industries.

### 3.3. Antidiabetic Activty

Amaranth seeds have demonstrated significant antidiabetic activity, with the underlying mechanism involving the inhibition of the enzymes dipeptidyl peptidase-IV (DPP-IV), α-glucosidase, and α-amylase by amaranth hydrolysates (AGs) and peptides (APs) [69,128]. DPP-IV is an enzyme that deactivates incretins, hormones essential for the secretion of insulin. This research suggests the potential of using amaranth peptides as functional food ingredients for diabetes prevention [69]. Kamal et al. [68] further demonstrated that amaranth protein hydrolysates, prepared using bromelain under in vitro conditions, exhibited enhanced inhibition of angiotensin-I-converting enzyme (ACE), DPP-IV, and α-glucosidase compared to amaranth protein isolate and other protein hydrolysates.

According to findings by Yang et al. [129], amaranth may contribute to improved metabolic health by modulating the composition of the intestinal microbiota. This includes increased bacterial diversity, a rise in beneficial bacteria like *Bifidobacteriaceae* and butyrate-producing bacteria (*Lachnospiraceae* and *Ruminococcaceae*), and a reduction in *Porphyromonadaceae*. These changes could lead to improved hepatic lipid metabolism and potentially enhance insulin sensitivity, possibly through alterations in metabolic pathways and reduced inflammation [129]. Olagunju et al. [130] developed amaranth-based multigrain bars with low and medium glycemic indices, which help prevent rapid fluctuations in blood glucose levels. Consumption of these bars did not result in significant increases in glucose levels in the subjects, indicating their potential benefit for glycemic control. Additionally, these bars exhibited considerable antioxidant activity, including the ability to neutralize radicals and bind metals, which may help combat oxidative stress—a common issue for diabetics [130]. Other studies have shown that multigrain snacks containing amaranth have low to medium glycemic indices, which help to reduce postprandial glucose and insulin levels, inhibit the activity of carbohydrate-hydrolyzing enzymes, improve antioxidant status, and reduce oxidative stress [140]. The snack with 90% amaranth content proved to be particularly effective, highlighting its potential for inclusion in the diets of individuals with diabetes and metabolic disorders.

However, the study was conducted on a relatively small sample, which included 38 healthy, non-smoking, non-diabetic volunteers [130], as well as 40 diabetic patients divided into four groups of 10 individuals, and 10 non-diabetic participants [140]. This underscores the need for further confirmation of these findings through larger-scale clinical trials. Additional studies involving a broader and more diverse participant pool will help refine the current results and enhance their practical significance, which is essential for the continued development of this research area.

### 3.4. Anticancer Activity

Cereal products, a significant source of dietary fiber, are rich in phenolic compounds, phytoestrogens, antioxidants, vitamins, and minerals that may have cancer-prevention properties [164]. Amaranth, like other pseudocereals, has emerged as a promising medicinal agent in the fight against cancer [131]. Plants in the *Amaranthaceae* family contain amino acid-balanced proteins that are essential sources of anticancer inhibitors.

A study by Maldonado-Cervantes et al. [165] identified a lunasin-like peptide (AhLun) in amaranth seeds with anticancer properties. The average concentration of AhLun was found to be 11.1 µg lunasin equivalent per gram of total extractable protein in four genotypes of mature amaranth seeds. AhLun can selectively induce apoptosis in tumor cells and requires less time to internalize into the nucleus of NIH-3T3 (mouse embryonic fibroblast) cells compared to soybean lunasin [165]. The molecular mechanisms behind AhLun’s chemopreventive properties were reviewed by Mazorra-Carrillo et al. [132]. Among the protein fractions, the glutelin fraction contained the highest concentration of lunasin (3.0 µg/g). Lunasin was also identified in the albumin, prolamin, and globulin fractions of amaranth protein [166].

These anticancer properties are further supported by the studies of Mazorra-Carrillo et al. [132], which found that the addition of amaranth AhLun to the culture medium did not alter cell morphology but significantly reduced the formation of anisokaryosis in cells treated with the carcinogen 3-methylcholanthrene (3MC). Furthermore, AhLun can influence the accumulation of tropomyosin, a protein considered a cancer biomarker. By regulating RNA-binding FuS (fused in sarcoma), vimentin, and profilin, AhLun can control cell shape and organelle movement. This study additionally found that the Serpin H1 protein, which is associated with a delay in mitosis during the G1/S phase, accumulated in the presence of lunasin, suggesting an additional mode of action for amaranth protein as a biologically active tumor suppressor compound [132].

A study by Taniya et al. [133] demonstrated that bioactive peptides from amaranth seed protein hydrolysates induce apoptosis and exhibit anti-migration effects in breast cancer cells. Thermally denatured hydrolysates from amaranth seed proteins (ASP-HDs) have been shown to enhance the absorption and release of essential amino acids and peptides that possess antioxidant activity and the ability to neutralize free radicals. In a study, human triple-negative breast cancer cells were cultured with varying concentrations of ASP-HD. The results of the experiment demonstrated that treatment with ASP-HDs inhibited the growth of cancer cells in a concentration-dependent manner, with a GI50 value of 48.3 ± 0.2 µg/mL. Additionally, the morphology of cells treated with ASP-HDs was examined, revealing significant changes, including membrane disruption, a decrease in cell number, and swelling, similar to what is observed with curcumin treatment (positive control). In control cells, over 50% migration was observed within 24 h, while in cells treated with ASP-HDs, this value was less than 10%, demonstrating the antimetastatic effects of amaranth peptides [133].

In addition to peptides, amaranth seeds contain significant amounts of squalene, which also exhibits antitumor properties [141,142]. Research findings indicate that squalene can inhibit the growth of cancer cells, stimulate apoptosis, influence metastasis, enhance the efficacy of chemotherapy drugs (such as gemcitabine), and impact signaling pathways related to DNA damage [167,168,169].

Phytol and α-tocopherol, found in amaranth, have demonstrated potential effectiveness in combating prostate cancer, as confirmed by molecular docking methods [170]. Docking results showed that α-tocopherol has the highest binding energy with AKR1C3 (aldo-keto reductase family 1 member C3), indicating its potential to inhibit this enzyme and possibly affect the control of tumor cell growth. Phytol also demonstrated significant effects, second only to α-tocopherol.

### 3.5. Antihypertensive Effect

Research and clinical trials have shown that primary risk factors for high blood pressure include external conditions, such as dietary and lifestyle factors like sodium and potassium intake, alcohol consumption, body weight, physical activity levels, socioeconomic status, and genetics. Improving these factors can help reduce the risk of developing hypertension [171].

Recently, amaranth has attracted attention due to its peptides, which can inhibit angiotensin-converting enzyme (ACE) [172]. Amaranth powder hydrolysates also exhibit antihypertensive effects by inhibiting ACE. ACE is responsible for activating the vasoconstrictor (angiotensin-II decapeptide) and degrading the vasodilator (bradykinin), leading to increased blood pressure. Therefore, ACE inhibition is one of the established approaches for treating hypertension [173].

The introduction of isolates and hydrolysates containing peptides from *A. hypochondriacus* into spontaneously hypertensive rats over a 3 h period resulted in a reduction in blood pressure from 200 to 220 mmHg to 140–170 mmHg [143].

Daskaya-Dikmen et al. [173] identified 116 peptides from amaranth, 17 of which are predicted to be bioactive, with 6 bioactive peptides exhibiting a high ACE inhibitory potential.

In the studies by Cruz-Casas et al. [135], 125 new peptides were identified from amaranth hydrolysates, of which 10 demonstrated significant ACE inhibitory potential. Additionally, fermentation using the *E. faecium*-LR9 strain was found to be a promising method for obtaining bioactive peptides such as IFQFPKTY and VIKPPSRAW, which exhibit strong antihypertensive effects.

In a study by Santiago et al. [134], a test was conducted on the digestion of an amaranth seed beverage using the Dynamic Digestion DIDGI^®^ system, which comprises gastric and intestinal compartments, to evaluate its ACE inhibitory activity. The gastric contents obtained after 105 min of digestion demonstrated a high ability to inhibit ACE (around 90% inhibition, IC50 = 80 ± 10 µg protein/mL), which was higher than the full digestion process (around 85% inhibition, IC50 = 140 ± 20 µg/mL) [134]. These data suggest that amaranth is a source of antihypertensive peptides after digestion, which are further hydrolyzed in the intestine, leading to the formation of new peptides with lower activity compared to those found in the gastric compartment.

The introduction of isolates and hydrolysates containing peptides from *A. hypochondriacus* into spontaneously hypertensive rats for 3 h resulted in a reduction in blood pressure from 200 to 220 mmHg to 140–170 mmHg [134]. In the group of rats fed amaranth cookies, blood pressure significantly decreased compared to those receiving water and control cookies (*p* < 0.05). This was also linked to ACE, whose activity was inhibited by the active compounds in amaranth hydrolysate. Moreover, cookies enriched with amaranth hydrolysate and captopril showed similar antihypertensive effects (*p* > 0.05), which persisted for 6 and 7 h, respectively (*p* < 0.05 compared to the control group of cookies and water).

Ontiveros et al. [144] reports that squalene, found in amaranth seeds, also exhibits a preventive effect in hypertension and ischemic heart disease. Accordingly, amaranth may be utilized as a functional food to help lower blood pressure in individuals with hypertension.

### 3.6. Antimicrobial Properties

Consumers today are increasingly cautious about chemical antibiotics due to their side effects on health. Natural antimicrobial agents are gradually gaining attention as an alternative to antibiotics for controlling foodborne pathogens [174]. Amaranth (*Amaranthus* spp.) is not only known as a nutritious source of proteins and antioxidants but also as a promising agent with antimicrobial properties [175]. Studies have shown that protein isolates from amaranth seeds possess a significant ability to suppress the growth of pathogenic microorganisms, including *Candida albicans*. Specifically, in vitro analysis indicates that amaranth proteins inhibit the transition of *C. albicans* yeast cells into their virulent hyphal form, a process that plays a crucial role in the development of infections. The antimicrobial activity of amaranth seeds is attributed to biologically active compounds, including polyphenols and flavonoids, which possess both antioxidant and antimicrobial properties.

The antioxidant activity of polyphenols may paradoxically induce oxidative stress in *C. albicans* by generating reactive oxygen species (ROS) at high concentrations. This disrupts cellular redox balance, impairing metabolic activity and viability, as evidenced by the XTT assay showing reduced cell viability at 50 μg/mL. In zebrafish models, it was found that amaranth protein isolates prevent yeast cell adhesion to the embryos’ membranes, thus protecting them from infection [145]. The specificity for biofilm and hyphal inhibition, without fungicidal effects, highlights the potential of amaranth peptides as safe and natural food supplements for preventing *C. albicans* infections in immunocompromised individuals.

### 3.7. Immunoregulatory Properties

Inflammation is a complex and dynamic biological process that plays a key role in protecting the body from damage, infections, and various forms of stress [176]. Amaranth, especially its peptides released during simulated digestion, has demonstrated significant immunoregulatory properties, highlighting its potential as a natural source of bioactive compounds with anti-inflammatory and antioxidant effects. A study by Sandoval-Sicairos et al. [146] on germinated amaranth flour (GAF) subjected to simulated gastrointestinal digestion (SGD) demonstrated that the peptides released during digestion, particularly those with a molecular weight of less than 10 kDa, exhibit potent anti-inflammatory effects. Inducible nitric oxide synthase (iNOS) in RAW 264.7 cells produces nitric oxide (NO), which plays a crucial role in the body’s defense mechanisms by targeting and destroying tumor cells, viruses, and bacteria. However, excessive production of NO leads to tissue damage and contributes to the development of inflammatory diseases [177]. These peptides were found to reduce NO production in lipopolysaccharide (LPS)-induced RAW 264.7 macrophages. The most substantial anti-inflammatory effect was observed in the 90 min digestion fraction (GAD90), which showed a significant reduction in NO levels, ranging from 42.0% to 53.0% lower compared to the positive control [146]. This suggests that peptides in the GAD90 fraction have a more potent anti-inflammatory effect than those in earlier digestion fractions (10–60 min), possibly due to the higher concentration of bioactive peptides released at this stage.

The study showed that smaller peptides, particularly those in the 3–10 kDa fraction (F2), exhibit the strongest antioxidant activity, likely due to the presence of aromatic amino acids such as tyrosine and phenylalanine. Furthermore, it was found that amaranth peptides possess various bioactive properties, including the potential to inhibit pro-inflammatory mediators and modulate the inflammatory response, which is necessary for regulating immune function. These findings align with previous studies conducted by Moronta et al. [147], which showed that amaranth demonstrates significant immunoregulatory properties, primarily due to bioactive peptides released during hydrolysis.

In a study on the effects of amaranth hydrolysates on activated human epithelial cells, it was found that these peptides inhibit the activation of Caco-2 cells induced by flagellin (FliC), a model for intestinal inflammation. The hydrolysates reduced the production of the chemokine CCL20 and blocked nuclear factor κB (NF-κB) activation, a key inflammatory pathway, in a dose-dependent manner. Among the identified peptides, SSEDIKE (Peptide 2) and IADEDPDEANDK (Peptide 3) exhibited strong anti-inflammatory effects, mainly when used in combination. Inhibition of the NF-κB signaling pathway, as well as the reduction in inflammatory markers such as cyclooxygenase-2 (COX-2) and inducible nitric oxide synthase (iNOS), suggests that amaranth peptides may be beneficial for treating inflammatory diseases. These results highlight the potential of amaranth as a functional food ingredient with immunomodulatory properties, which could benefit both healthy individuals and those suffering from inflammatory diseases such as inflammatory bowel disease [147].

### 3.8. Hypocholesterolemic Effect

Amaranth has been shown to have the potential to lower cholesterol levels by inhibiting key enzymes involved in fat digestion and absorption [136]. The high fiber content and presence of sterols in amaranth contribute to cholesterol reduction by preventing its absorption into the bloodstream [178,179]. Amaranth protein hydrolysates (APHs), especially those obtained using bromelain, chymotrypsin, and actinase E, have shown the ability to inhibit the activity of two critically important enzymes: cholesterol esterase (CEase) and pancreatic lipase (PL) [136]. Cholesterol esterase plays a role in the hydrolysis of cholesterol esters in the intestinal lumen. At the same time, pancreatic lipase is responsible for breaking down triacylglycerols into free fatty acids and mono-glycerides, which are then absorbed by intestinal epithelial cells. By inhibiting these enzymes, amaranth hydrolysates reduce the absorption of cholesterol and fat, potentially leading to a decrease in plasma cholesterol levels [148].

In one study [136], hydrolysates obtained using bromelain, particularly those hydrolyzed for 4–6 h, demonstrated strong inhibitory activity against cholesterol esterase and pancreatic lipase, with IC50 values ranging from 0.47 to 0.53 mg/mL for cholesterol esterase and from 0.38 to 0.66 mg/mL for pancreatic lipase. These results suggest that amaranth peptides may act similarly to commercial lipase inhibitors, making them a promising natural remedy for managing hypercholesterolemia. The inhibition of cholesterol esterase and pancreatic lipase by amaranth peptides is attributed to their ability to bind to critical amino acid residues in the enzymes’ active sites, thereby disrupting their catalytic function. Peptides identified in the hydrolysates, such as FPFVPAPT, MPFLPR, and FPFVGP, showed significant binding potential to the active sites of both cholesterol esterase and pancreatic lipase. The presence of hydrophobic amino acids, such as leucine and proline, in these peptides suggests their effective interaction with the enzymes, blocking their activity [149]. This mechanism of action aligns with the observed hypocholesterolemic effects of amaranth, which have been further supported by previous studies showing reduced cholesterol levels in animal models [149].

Soares et al. [150] investigated the cholesterol-lowering effect of peptides derived from the hydrolysis of amaranth protein (*A. cruentus*). The researchers identified specific peptides, such as GGV, IVG, and VGVL, that can inhibit 3-hydroxy-3-methyl-glutaryl-CoA reductase (HMG-CoA), a key enzyme in cholesterol synthesis, indicating their potential as new natural hypocholesterolemic agents. Therefore, amaranth hydrolysates can be utilized as functional ingredients in foods designed to lower cholesterol and enhance cardiovascular health.

### 3.9. Weight Management and Satiety (Anti-Obesity)

Obesity is one of the leading causes of cardiovascular diseases, including hypertension, atherosclerosis, stroke, heart failure, and arrhythmias, particularly atrial fibrillation and sudden cardiac death [180]. Thus, dietary interventions, including functional food components, are promising therapies for improving obesity-related conditions and warrant further investigation [181].

A study presented by Wu et al. [137] demonstrates that dietary interventions incorporating amaranth compounds, particularly those rich in lutein, anthocyanins, and saponins, can mitigate weight gain induced by a high-fat diet in mice. After 12 weeks of treatment, these compounds significantly reduced body weight compared to mice on a high-fat diet, with the best results observed in the groups enriched with anthocyanins and saponins. Weight reduction was accompanied by improvements in metabolic parameters, including decreased levels of triglycerides, total cholesterol, and low-density lipoprotein (LDL) cholesterol, which are associated with obesity and its complications. Additionally, amaranth compounds have been shown to have a positive effect on glucose metabolism, significantly improving glucose tolerance and reducing blood sugar levels [137]. Further studies have shown that amaranth compounds also alleviate dyslipidemia, as evidenced by a reduction in triglyceride and cholesterol levels in the serum and liver of obese mice. These compounds affect key genes involved in lipid metabolism, such as peroxisome proliferator-activated receptors (PPARα and PPARγ) and fatty acid synthase (FAS), suggesting that amaranth can regulate lipogenesis and lipolysis in adipose and liver tissues [137]. This makes amaranth a promising natural functional product that can play a role in preventing and treating obesity, offering a multifaceted approach to combating obesity-related diseases.

## 4. Innovative Applications of Amaranth in Functional Foods and Nutraceuticals

**Figure 3:**
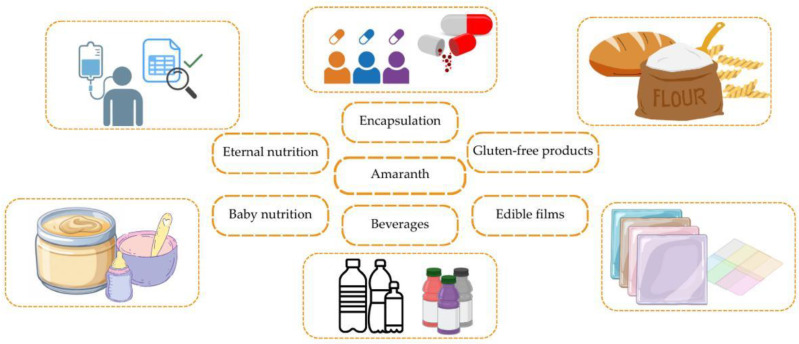
Innovative products based on amaranth grain.

Amaranth, with its unique nutritional composition and versatile application as a superfood, is widely used in the development of functional food products and specialized nutrition [182]. Moreover, amaranth grain represents a promising ingredient with numerous potential health benefits [183]. The study by Kumar et al. [25] covers various aspects of using amaranth grain in the development of innovative food products. Promising applications of amaranth in functional foods and nutraceuticals are presented in Table 3 and Figure 3.

### 4.1. Gluten-Free Products

Amaranth differs from traditional grains in that it contains only a small amount of prolamins, making it safe for people with celiac disease or gluten sensitivity [139]. As noted in the previous section, the predominant albumins, globulins, and glutelins in amaranth’s protein composition do not cause allergic reactions in most people with gluten intolerance [117]. Due to its low prolamin content and high quality of other proteins, amaranth is an excellent alternative for gluten-free diets [139].

In the study by Woo et al. [184], the effect of sourdough made from amaranth protein isolate on the quality of gluten-free bread made from buckwheat flour was evaluated. The duration of fermentation played a significant role in the final product’s quality. The study demonstrated that the addition of amaranth protein isolate facilitated the formation of a protein network, particularly at temperatures above 70 °C, resulting in the generation of smaller protein fragments. Extended fermentation substantially altered the protein structure, which positively impacted the rheological and structural properties of the dough, improving the quality of the finished gluten-free bread [184].

Amaranth proteins are characterized by good solubility, high emulsifying and foaming abilities, and the ability to form gels, which, along with excellent thermal stability, make amaranth a valuable ingredient for gluten-free product manufacturing [139]. Vici et al. [185] noted that people with celiac disease often have a deficiency in folic acid. Their research showed that the consumption of enriched bread, particularly bread made with amaranth sourdough and non-encapsulated spirulina, had high folate content (4154.42 µg of folic acid equivalents per 100 g of fresh weight) among all the bread samples tested [194].

In the development of the optimal blend of composite flours for gluten-free products, it was found that a mixture consisting of 50% amaranth flour, 40% buckwheat flour, and 10% quinoa was effective for producing various gluten-free products [195].

The demand for plant-based dairy products has continued to increase steadily among populations worldwide [196]. The production of yogurt made from sprouted buckwheat and amaranth has the potential for wide application in gluten-free products. Studies show that integrating grains and sprouted pseudo-grains into yogurt substitute formulations presents opportunities for creating innovative products with enhanced nutritional properties and potential health benefits [196].

To expand the range of gluten-free products, a study was conducted on amaranth pasta, which found that amaranth, as a natural gluten-free product with high nutritional value, is a promising ingredient for producing gluten-free pasta [197]. The research also demonstrated that amaranth pasta retains its shape even after extended cooking times of up to 15 min. Gluten-free cookies made with a composite flour of amaranth seeds and tiger nuts yielded acceptable and comparable results in terms of appearance, taste, aroma, and overall evaluation when compared to traditional wheat cookies [198].

During the study, an extrusion technology was developed for producing snacks using improved blends of amaranth flour, soybean meal, and shallot flour, resulting in nutritious and functional foods. The optimized mixture enabled the creation of extruded snacks with excellent functional characteristics, including high antioxidant activity, low levels of antinutrients, a low glycemic index, and good nutritional properties [199].

### 4.2. Baby Nutrition

Adequate baby nutrition remains a top priority in healthcare and food security, as it significantly affects a child’s growth, development, and immune system strengthening [200]. A quantitative study conducted as part of the Comprehensive Nutritional Rehabilitation Program (CNRP) demonstrated that the use of amaranth flour as supplementary nutrition for children with mild malnutrition led to statistically significant improvements in anthropometric indicators, including body weight, upper arm circumference, subscapular skinfold, and triceps skinfold [186].

Amaranth-containing products helped increase nutrient intake among children aged 6–23 months, and a more significant number of children were able to meet the recommended daily intake of essential nutrients [187]. The use of amaranth grain in infant nutrition is due to its high content of arginine and histidine [60].

Jiménez et al. [188] investigated the oxidative stability of infant dried purees prepared with various oils and sprouted quinoa and amaranth grain flours. The sprouting process had different effects on the tocopherol content in quinoa and amaranth: the tocopherol level decreased in quinoa, while it increased in amaranth. Purees made from sprouted grain flour exhibited better oxidative stability, despite an increase in unsaturated fatty acids, which was attributed to the higher tocopherol content.

### 4.3. Nutraceuticals

Amaranth has a range of potential nutraceutical benefits due to its rich nutritional composition and content of bioactive compounds [189]. The bioactive compounds in amaranth exhibit pronounced antioxidant properties, reducing oxidative stress in the body and positively impacting physiological health [190]. Numerous studies have shown that amaranth grain, particularly its proteins and encapsulated peptides, has cardioprotective effects [191].

### 4.4. Amaranth-Based Beverages

Functional beverages represent a rapidly growing market segment, serving as an effective matrix for delivering nutrients and bioactive compounds [201]. In recent years, amaranth-based beverages have attracted growing interest from researchers as a promising source of natural antihypertensive compounds. Contemporary studies highlight their potential for the development of functional foods and nutraceuticals aimed at the prevention of arterial hypertension.

A study by Suárez et al. [134] demonstrated that amaranth proteins, incorporated into a high-protein beverage, become an effective source of peptides with significant antihypertensive activity after undergoing simulated gastrointestinal digestion. The final digestion product exhibited strong angiotensin-converting enzyme (ACE) inhibitory activity, confirming its functional potential.

Moreover, the study identified 26 peptide sequences, some of which contained fragments previously recognized for their antihypertensive properties. The presence of such fragments in the structure of amaranth proteins indicates their inherent capacity to generate physiologically active peptides during enzymatic hydrolysis [134].

When preparing beverages, amaranth seeds are initially ground together with hydrocolloids using a colloid mill, followed by centrifugation and filtration. These technological steps play a key role in determining the quality characteristics of the final product [134,192,193]. Denaturation and aggregation of proteins was caused by thermal processing, whereas the addition of gum reduced the sensitivity of protein to heat treatment, helping to preserve their functional properties [192].

Protein hydrolysates obtained through the fermentation of amaranth by lactic acid bacteria (LAB) and *Bacillus* species demonstrated antioxidant, antihypertensive, and antimicrobial activity. These bioactive properties indicate the potential use of hydrolysates in functional foods and nutraceuticals for disease prevention [202]. Compared to amaranth flour, processing amaranth grain into a beverage has a complex effect on the product’s overall antioxidant potential. On one hand, beverage processing may lead to potentially adverse effects, such as moderately increased cytotoxicity in non-dialyzed samples, whereas amaranth flour has been associated with a significant increase in glutathione levels [193].

However, after simulating gastrointestinal digestion, beverages made from amaranth show a strong antioxidant potential despite these limitations. Their capacity to suppress intracellular ROS, activate superoxide dismutase, and exhibit greater ORAC activity compared to amaranth flour serves as evidence of this process.

Therefore, amaranth beverages offer a novel approach to the creation of antioxidant-rich functional foods that could help control blood pressure and preserve cardiovascular health [193].

### 4.5. Encapsulation of Bioactive Compounds

Recent growing interest in functional foods has driven research into creating new methods for encapsulating bioactive components [203,204,205]. An auspicious direction is the use of natural biopolymers as coating materials through complex coacervation, providing protection and transport for nutraceuticals sensitive to environmental factors [206,207,208,209].

The use of biopolymers from amaranth grain, specifically starch and protein fractions, through spray drying as coating materials for encapsulating β-carotene enables the creation of stable and biologically valuable products that retain their properties during storage and digestion [210]. First of all, the obtained microcapsules are characterized by high encapsulation efficiency (64–69%), which ensures a significant concentration of β-carotene and other bioactive compounds in the shell. Secondly, protein matrices form a dense three-dimensional network structure that acts as a physical barrier against thermal and photonic exposure. As a result, protein-based microcapsules retain significantly more β-carotene when stored at 8 °C and 25 °C, especially in the absence of light, compared to starch-based analogs. Thirdly, during in vitro digestion modeling, the release of β-carotene is regulated by the mechanism of structural relaxation of the polymer shell at pH 7.4 and 37 °C, which enhances the bioavailability of the encapsulated compound compared to its free form. Finally, amaranth protein possesses antioxidant activity and is capable of forming molecular complexes with β-carotene, which further prevents its oxidative degradation both during storage and technological processing [210].

In studies by Constantino et al. [211,212], innovative microencapsulation systems for betanin and β-carotene were developed using a combination of ultrasound-isolated amaranth protein and carboxymethyl cellulose, as well as carboxymethyl starch from amaranth and lactoferrin. Encapsulated betanin demonstrated 2.92-times-higher heat resistance compared to free betanin, with 84% of the encapsulated betanin retaining its bioavailability. Encapsulated β-carotene also showed a significant increase in heat resistance, though its bioavailability was 27.92%. The microcapsules effectively protected both betanin and β-carotene in the acidic environment of the stomach, confirming their high stability and functionality in the digestive tract.

Adding encapsulated betanin to gelatin films helped reduce light transmittance, increase antioxidant activity, and improve the mechanical properties of the films, including flexibility. Similarly, adding encapsulated β-carotene to jelly candies led to improved textural characteristics, including reduced hardness, decreased chewiness, and reduced stickiness. At the same time, important properties such as adhesiveness, elasticity, and resilience were maintained, and the appearance of the candies remained visually appealing, highlighting their potential for use in products with enhanced functional properties [211,212].

Microencapsulation of vitamin D3 using heteroprotein complexes formed from amaranth protein isolates and lactoferrin achieves high encapsulation efficiency, reaching up to 90%. This method effectively protects vitamin D3 from degradation caused by environmental factors, such as ultraviolet radiation and high temperatures. Additionally, this technology promotes the controlled release of vitamins in the digestive tract, making it a promising approach for use in functional foods and fortified baked goods [213].

### 4.6. Edible Coating or Active Films for Food Preservation

Recently, researchers focused on the development of biodegradable films have turned their attention to enhancing their properties by incorporating new functional characteristics [100]. This approach addresses several challenges related to packaging quality and extending the shelf life of food. One of the most promising directions in this field is the development of active films that provide additional protective and technological advantages [214].

Amaranth-based films exhibit excellent barrier properties against water vapor and high film-forming characteristics [215]. Studies by Tapia-Blácido et al. [216] and Chandla et al. [217] demonstrated that films made from amaranth flour possess excellent barrier properties against water vapor and good film-forming characteristics; however, their mechanical properties are inferior to those of synthetic packaging. Morales-Olán et al. [218] modeled the optimal composition of a film with chitosan nanoparticles (4% amaranth flour, 25% glycerol, and 0.36% chitosan nanoparticles). This achieved satisfactory permeability for water vapor, tensile strength, and antioxidant activity. In the study by Coelho et al. [219], a new combination of materials was explored to create edible and eco-friendly packaging materials based on alginate films with amaranth extract. The results showed that such films exhibited increased antioxidant activity and enhanced opacity, which was linked to the addition of phenolic compounds.

Edible films made from modified amaranth starch subjected to thermo-humid processing demonstrated improved functional properties. The permeability of the films to water vapor decreased, enhancing their barrier characteristics and reducing moisture loss. Mechanical properties, such as tensile strength and elasticity, also improved significantly, making the films more resistant to external factors and expanding their potential use in food packaging [220]. Das et al. [139] noted the potential use of amaranth protein in the food and packaging industries. Adding amaranth protein to biopolymer films improves their optical properties and mechanical characteristics.

### 4.7. Potential Application in Enteral Nutrition

Clinical nutrition plays a crucial role in modern medicine by providing patients in critical conditions with essential nutrients and energy according to their daily requirements [221]. In recent years, there has been significant interest in developing enteral nutrition based on plant-based raw materials [222,223,224]. Existing sources indicate a significant interest from organizations such as EAT-Lancet, The Good Food Institute, and Protein2Food in plant-based protein products [225,226]. According to ASPEN recommendations, the daily requirement for adults is 20–35 kcal/kg [227]. For elderly patients experiencing metabolic stress, a protein intake of 1.2–2.0 g/kg is typically required [228,229].

Numerous studies have concluded that plant-dominant protein blends used in enteral nutrition do not coagulate in the stomach, promote faster gastric emptying, are better tolerated by patients, and result in smaller volumes of residual contents in the stomach compared to enteral nutrition based on animal proteins [225,230,231]. Plant-based enteral nutrition helps maintain a higher level of intestinal microbiota diversity, increases the concentration of beneficial metabolites, and stimulates the growth of commensal bacteria such as *Clostridiales*, *Lachnospiraceae*, and *Ruminococcus*, which are associated with maintaining gut health. Additionally, it helps reduce pathogenic microorganisms, positively affecting the microbiota and the overall health of the body [222].

Among the many studied crops, amaranth has been shown to have a positive effect on the intestinal microbiota [114], exhibits potential antidiabetic activity [113], and is rich in micronutrients, dietary fibers, vitamins, minerals, and other bioactive components, including polyphenols, phytosterols, carotenoids, and squalene [22,117,126,139,232]. Amaranth grain is a good source of protein characterized by low allergenicity and good tolerance in most people with gluten intolerance [117]. Furthermore, its increased content of high-quality protein, with a complete and balanced composition of essential amino acids [42] and unsaturated fatty acids [69], makes it a valuable raw material for enteral nutrition. To improve protein balance in critically ill patients, the use of amaranth protein as a component of enteral nutrition is being considered [233]. Pavlik et al. [233] noted that amaranth protein aids in recovery during illness and surgery, compensating for protein deficiencies in patients with various degrees of malnutrition. Due to its high lysine and tryptophan content, amaranth protein exhibits properties similar to those of animal proteins [234], which is important for enteral nutrition. Unsaturated fatty acids help lower cholesterol levels, reducing the risk of atherosclerosis and related complications [235].

Enteral nutrition, which contains the necessary amount and quality of protein, is an effective means of preventing weight loss, reducing gastrointestinal side effects, ensuring adequate nutrition, and preventing allergic reactions in patients. Plant-based enteral nutrition can also be used for patients with specific dietary requirements [236,237]. Enteral formulas characterized by a low glycemic index and high nutritional value, especially those with increased protein and fiber content [238], are particularly beneficial.

Therefore, amaranth is an excellent candidate for the development of new enteral formulas that not only effectively provide patients with the necessary nutrients but also have a positive impact on their health. Further research in this area presents a promising direction that could lead to the creation of new clinical nutrition products that enhance the quality of life for patients.

### 4.8. Limitations of Amaranth Grain Utilization

Despite its rich nutritional profile and promising functional and therapeutic attributes, amaranth grain remains underutilized in the food industry due to several factors that require further research. The presence of antinutrients, including nitrates, oxalates, phytates, saponins, and tannins, can diminish the bioavailability of essential micronutrients and impart a bitter taste, subsequently limiting its applications [29,239]. In recent years, innovative processing techniques, such as cold plasma, high hydrostatic pressure, irradiation, pulsed electric fields, pulsed light, and ultrasonic processing, have emerged as potential solutions to mitigate these antinutrients [240,241]. These technologies aim to reduce the antinutrient content, thereby improving the nutritional and functional properties of amaranth and increasing its potential use in food products. Nevertheless, clinical studies have already demonstrated some of the beneficial properties of amaranth, and further research is needed to fully understand its therapeutic effects and their long-term impact on human health.

## 5. Conclusions

Amaranth (*lat. Amaranthus*) is a pseudocereal crop with high nutritional and biological value. Amaranth grain is characterized by a high protein content, up to 21.5%, which significantly exceeds the corresponding figure for most traditional cereal crops. The protein profile of amaranth includes all essential amino acids, with particularly high concentrations of lysine, methionine, cysteine, and tryptophan. This makes amaranth a valuable source of complete protein, especially within vegetarian diets and gluten-free diets.

The lipid composition of the grain varies from 6 to 9%, and in some species, it can reach up to 19%. The main portion of fats consists of unsaturated fatty acids (up to 83%), including linoleic, oleic, and palmitoleic acids. Notably, the content of the biologically active compound squalene (up to 11% of the total lipid fraction), as well as tocopherols with antioxidant activity, is remarkable.

Amaranth grain is a source of dietary fiber, vitamins (B, C, and E), and minerals—potassium, calcium, magnesium, phosphorus, iron, and zinc. The composition also includes various bioactive compounds, including flavonoids (quercetin, kaempferol, rutin, vitexin, isovitexin) and phenolic acids (ferulic, gallic, vanillic, etc.).

Modern studies confirm the antioxidant, antihypertensive, antitumor, hypocholesterolemic, immunomodulatory, antidiabetic, and antimicrobial activities of amaranth components. One of the key areas is the positive impact of amaranth on the composition of the intestinal microbiota and the enhancement of short-chain fatty acid production, which contributes to the improvement of the body’s metabolic status and the reduction in systemic inflammation.

Clinical and preclinical data indicate the effectiveness of amaranth peptides in inhibiting enzymes involved in the pathogenesis of diabetes, hypertension, and hypercholesterolemia. In particular, the ability of amaranth peptides to inhibit DPP-IV, α-glucosidase, pancreatic lipase, cholesterol esterase, and ACE has been established.

Moreover, amaranth peptides and other compounds exhibit high selective activity against tumor cells by modulating signaling pathways associated with cell proliferation, apoptosis, and inflammation.

Amaranth has also shown immunomodulatory properties, manifested in the ability of its components to reduce the production of pro-inflammatory markers, inhibit inflammatory signaling pathways (NF-κB), and enhance the body’s resistance to infectious and metabolic stresses. It has been proven that amaranth peptides can be used as natural anti-inflammatory and antimicrobial agents, especially in conditions of impaired immune response.

Studies demonstrate amaranth as a safe and highly nutritious component for gluten-free diets, and confirm its ability to provide improved qualities in products such as bread, pasta, snacks, and baby food. Moreover, its bioactive compounds, including antioxidants and polyunsaturated fatty acids, open up prospects for the use of amaranth in nutraceuticals and functional beverages.

Amaranth is an effective source of protein with a complete amino acid profile, making it valuable for enteral nutrition, especially during the recovery period after illnesses or surgical interventions. Its low allergenicity, high nutritional value, and good tolerance in patients with celiac disease underscore its significance in specialized diets.

Promising methods of encapsulating bioactive compounds and developing edible packaging based on amaranth open new horizons for innovative solutions in the food and packaging industries.

Amaranth is characterized by high resistance to adverse conditions such as saline and alkaline soils, high temperatures, and moisture deficiency. This makes it promising for widespread cultivation, especially in the context of climate change. Its resistance to pests and the need for minimal use of chemical fertilizers make amaranth an environmentally friendly product, which increases its value against the backdrop of growing demands for environmentally safe agriculture.

Thus, all components of the amaranth grain, including proteins, lipids, bioactive compounds, and minerals, are closely related to its therapeutic properties. Functional properties, combined with a rich chemical composition, make amaranth grain promising for the development of specialized food products aimed at the prevention of various diseases and maintaining the nutritional status of patients on enteral nutrition.

## Figures and Tables

**Table 1:** Summary of essential amino acids in amaranth grain (mg/g).

	Composition (mg/g)			
Name of the Essential Amino Acid	[5]	[53]	[54]	[55]
Leucine	6.20	7.11	7.31	7.85
Valine	5.90	4.62	4.53	3.11
Lysine	5.70	8.97	5.59	10.10
Phenylalanine	5.40	6.55	6.84	3.80
Threonine	5.10	4.40	4.52	3.05
Methionine	4.60	3.03	3.35	4.69
Isoleucine	3.90	4.38	4.06	2.87
Histidine	3.00	4.08	3.98	6.83

**Table 2:** Clinical studies of amaranth grain impact on human health.

Name of the Illness	Part or Product of Amaranth Used in Clinical Study	Effect of Treatment	References
**Intestinal dysbiosis, inflammation and colitis**	Amaranth popcorn	Decreased numbers of *Alistipes putredinis*, *Bacteroides coprocola*, and *Bacteroides stercoris* Increased numbers of *Akkermansia muciniphila*, and *Streptococcus thermophilus bacteria*	[138]
**Oxidative stress**	Protein fractions of amaranth	Antioxidant activity aids in neutralizing free radicals	[139]
**Diabetes**	Amaranth hydrolysates and peptides	Inhibition of the enzyme dipeptidyl peptidase IV, α-glucosidase, and α-amylase	[128]
	Low glycemic amaranth-based multigrain bars	Antioxidant activity neutralizes free radicals and binds metals, preventing sharp fluctuations in blood glucose levels	[130]
		A low glycemic index, decreased postprandial blood glucose levels, and enhanced activity of antioxidant enzymes in serum, such as catalase, superoxide dismutase, and glutathione peroxidase, along with increased glutathione levels	[140]
**Cancer, tumor**	Amaranth lunasin-like peptide	Reduces the accumulation of tropomyosin, decreases the formation of anizokaryosis, controls cell shape, and regulates the movement of organelles	[132]
	Squalene	Stimulates apoptosis, affects metastasis, and enhances the action of chemotherapeutic agents	[141,142]
**Breast cancer**	Thermally denatured hydrolysates from amaranth seed protein	Inhibition of cancer cell growth, antimetastatic activity	[133]
**High blood pressure**	Amaranth powder hydrolysate	Inhibition of angiotensin-converting enzyme activity	[134,143]
	Amaranth cookies		[144]
**Pathogenic microorganism *C. albicans***	Protein isolates from amaranth grain	Prevention of yeast cell attachment due to the high content of polyphenols and flavonoids	[145]
**Inflammatory illnesses**	Sprouted amaranth powder	Reduction in nitric oxide production, exerting an anti-inflammatory effect	[146]
	Amaranth bioactive peptides	Modulation of the inflammatory response by decreasing the production of chemokine (CCL20) and blocking NF-kB activation.	[147]
**Hypercholesterolemia, dyslipidemia**	Hydrolysate of amaranth protein	Inhibition of cholesterol esterase and pancreatic lipase enzymes	[136,148,149]
		Inhibition of 3-hydroxy-3-methylglutaryl-CoA-reductase	[150]
**Obesity**	Amaranth compounds: lutein, saponin, and anthocyanin	Improvement in metabolic parameters, reduction in triglyceride and cholesterol levels	[137]

**Table 3:** Emerging applications of amaranth in functional foods and nutraceuticals.

Application Area	Product Type	Functionality	Authors
Functional food products	Amaranth-based products (flour, cereals, mixtures)	Nutrient enrichment, health improvement	[25,182,183]
Gluten-free products	Bread, pasta, cookies, gluten-free mixes	Alternative to gluten-containing products, improving digestion	[139,184,185]
Baby food	Baby formulas, purees, cereals based on amaranth	Supports growth and development, source of proteins and microelements	[186,187,188]
Nutraceuticals	Amaranth supplements, capsules, powders	Antioxidant properties, support for the cardiovascular system	[189,190,191]
Amaranth-based beverages	Fermented drinks, protein shakes	Reduced hypertension, improved digestion	[134,192,193]

## Data Availability

No new data were generated during the course of this review.

## Abbreviations

The following abbreviations are used in this manuscript:

TAG	Triacylglycerol
LEA	Late embryogenesis abundant
GBSSI	Granule bound starch synthase I
SSPs	Seed storage proteins
LDL	Low-density lipoprotein
HDL	High-density lipoprotein
IBS	Irritable bowel syndrome
SCFA	Short-chain fatty acid
AG	Amaranth hydrolysate
DPP-IV	Dipeptidyl peptidase—IV
AP	Amaranth peptides
ACE	Angiotensin-I-converting enzyme
AhLun	Lunasin-like peptide
NIH-3T3	Mouse embryonic fibroblast
3MC	3-methylcholanthrene
ASP-HD	Amaranth seed protein—heat-denatured
AKR1C3	Aldo-keto reductase family 1 member C3
GAF	Germinated amaranth flour
SGD	Stimulated gastrointestinal digestion
iNOS	Inducible nitric oxide synthase
LPS	Lipopolysaccharide
GAD90	90 min digestion fraction
COX-2	Cyclooxygenase-2
PL	Pancreatic lipase
APH	Amaranth protein hydrolysate
HMG-CoA	3-hydroxy-3methyl-glutaryl-CoA reductase
FAS	Fatty acid synthase
PPAR	Peroxisome proliferator-activated receptor
